# A Case Series of Surgical Resection of Anterior and Posterior Butterfly Glioma Grade 4 via a Minimally Invasive Keyhole Approach

**DOI:** 10.7759/cureus.33787

**Published:** 2023-01-15

**Authors:** Dorin Bica, Artsiom Klimko, Ion Poeata

**Affiliations:** 1 Neurosurgery, Enayati Medical City, Bucharest, ROU; 2 Physiology and Neuroscience, University of Medicine and Pharmacy "Carol Davila", Bucharest, ROU; 3 Neurosurgery, University of Medicine and Pharmacy "Grigore T. Popa", Iași, ROU

**Keywords:** butterfly glioblastoma surgery, butterfly glioma, keyhole interhemispheric neurosurgery, keyhole neurosurgery, extent of resection benefit, survival benefit, keyhole approach

## Abstract

Surgical resection of infiltrating glial neoplasms has proven to improve quality of life and confer a significant survival benefit. As accumulating evidence cements the role of surgery in grade 4 gliomas, there is a general trend to transition away from traditional large craniotomies to smaller ‘keyhole’ approaches, which aim to reduce the trauma and complication profiles associated with large exposures. A keyhole approach uses a small craniotomy positioned perfectly to reach at least all the target structures that a conventional approach would reach. We present a case series of operated butterfly gliomas grade 4 patients through keyhole approaches. All three operated patients have better survival than the literature biopsy groups. The resection of butterfly gliomas should be considered in selected cases. For some patients, it is feasible with the technology used nowadays, with improved quality of life and better survival prognosis.

## Introduction

Grade 4 gliomas are the most common and aggressive malignant brain tumors. In a small subset of patients, grade 4 gliomas can infiltrate along the corpus callosum into the contralateral hemisphere, and due to its resemblance to a butterfly on MRI, it is termed butterfly grade 4 glioma (bGG). Surgical resection of infiltrating glial neoplasms has proven to improve quality of life and confer significant survival benefits [1-3]. As accumulating evidence cements the role of surgery in grade 4 gliomas, there is a general trend to transition away from traditional large craniotomies to smaller ’keyhole’ approaches, which aim to reduce the trauma and complication profiles associated with large exposures. A keyhole approach uses a small craniotomy positioned perfectly to reach at least all the target structures that a conventional approach would reach.

By searching PubMed, Scopus, and Web of Science databases, we found very few studies that evaluated the application of a keyhole approach for the resection of bGG with long-term follow-up. Current studies serve to prove the technical feasibility of resecting bGGs in selected cases [4-7], but fewer consider keyhole approaches for this type of tumor [8]. Due to the rarity of this pathology, we furthermore hope to add to the current understanding of keyhole cytoreduction of bGG by identifying outcomes and clinical characteristics of patients with these tumours to better understand the therapeutic approach for bGG.

## Case presentation

We present three patients with a histopathological diagnosis of grade 4 glioma with bihemispheric involvement who underwent minimally invasive keyhole surgical cytoreduction. General data on the three patients are presented in Table 1.

**Table 1:** General operative and morphologic data of the operated patients. KPS: Karnofsky performance scale; EOR: Extent of resection; LOS: Length of stay.

Operative and tumor data	Patient 1		Patient 2	Patient 3
KPS score (pre/post-op)		80/100	80/90	90/90
Tumor volume (cm^3^)		14.44	20.08	39.65
EOR (%)		98.75	84.86	74
Residual tumor volume (cm^3^)		0.18	3.04	10.27
Bone flap dimensions (cm)		2.8/2.09	4.48/3.74	4.00/2.85
LOS (days)		5	5	5
Survival (months)		27	36	>19

**Figure 1:**
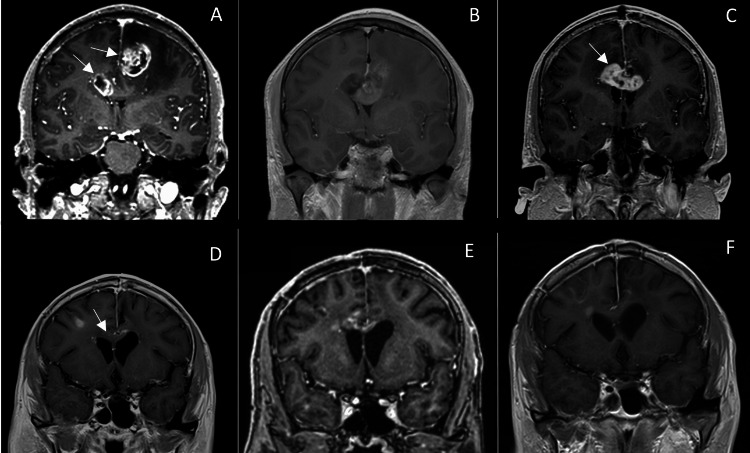
Follow-up MRIs of patient no. 1. (A) Preoperative image – bilateral enhancement suggesting bGG (arrows); (B) Day 1 postoperative image; (C) MRI at three months postoperatively with a contrast-enhanced lesion on the corpus callosum (arrows); (D) MRI at 18 months postop with excellent response and evolution due to oncological treatment with no enhancement on the corpus callosum (arrows); (E) MRI at 24 months after resection surgery; and (F) MRI at 27 months after resection surgery. bGG: Butterfly grade 4 glioma.

The first patient was a 43-year-old male who presented with episodes of intractable headache and vomiting. T1-weighted MRI revealed contrast-enhancing masses localized to the left and right frontal lobes, with a transcallosal connection between them visible on T2-weighted fluid-attenuated inversion recovery (FLAIR) images (Figure 1).

The patient was positioned in dorsal decubitus, and a keyhole left unilateral frontal interhemispheric approach was used. The 3 cm craniotomy was positioned over the superior sagittal sinus to allow for interhemispheric dissection and resection of the contrast-enhancing region in the frontal lobe until the corpus callosum was reached, where angulation was adjusted to resect the second contrast-enhancing focus via ultrasonic aspiration. The postoperative course was favorable, with the control MRI demonstrating an extent of resection (EOR) of 98.75%. The patient was able to return to work and experienced an excellent functional recovery. He survived for 27 months after surgery and passed away from complications precipitated by severe acute respiratory syndrome coronavirus 2.

**Figure 2:**
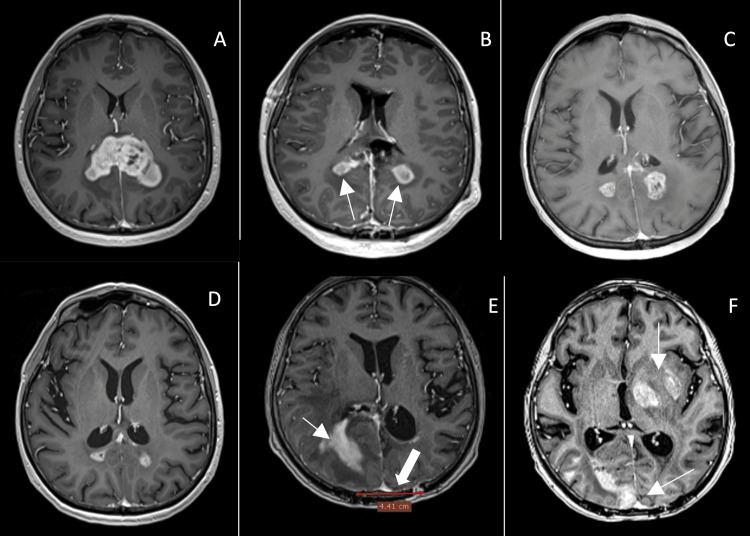
Follow-up MRIs of patient no. 2. (A) Preoperative image; (B) Day 1 postoperative image with RTV of 3 cm3 (arrows); (C) Four months postoperative image after radiotherapy and first temozolomide course; (D) Twenty months postoperatively; (E) Progression seen at 25 months postoperatively (arrow) and a maximum diameter of bone flap drawn on the image (thick arrow); and (F) Extended progression at 32 months postoperatively (arrows). RTV: Residual tumor volume.

The second patient was a 56-year-old female who presented with an intractable headache aggravated by recumbency. MRI revealed a mass localized to the splenium of the corpus callosum with bilateral extensions into the cerebral hemispheres (Figure 2).

**Figure 3:**
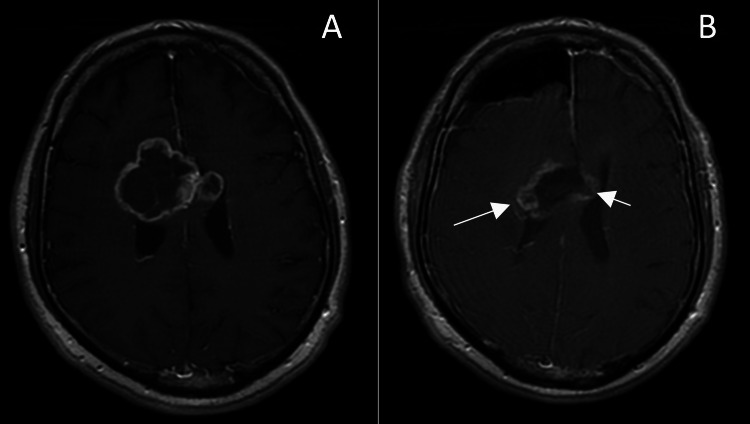
Follow-up MRIs of patient no. 3. (A) Preoperative T1-enhanced MRI image; (B) Day 1 postoperative image with residual tumor volume of 10.27 cm3 (arrows).

Diffusion tensor imaging (DTI) tractography was used to map the optic radiations to be included in operative planning. The patient was positioned in ventral decubitus, and a bilateral keyhole parieto-occipital approach was used. The craniotomy was positioned over the superior sagittal sinus to allow for interhemispheric excision of the invasion into the splenium of the corpus callosum and a minimal median parietal cortectomy. The excision continued laterally until the visualization of the lateral ventricles and anteriorly until the internal cerebral veins. Gross total removal (GTR) was not possible due to extensions of the tumor into the optic radiations. In the immediate postoperative period, the patient suffered from an episode of confusion and right-sided homonymous hemianopia, which completely resolved at the time of discharge four days after surgery. Transcranial stimulation electrophysiology was used for this patient. Postoperative MRI confirmed an EOR of 85%, with residual tumor volume further shrinking after chemoradiotherapy and remaining stable for 21 months. Progression is documented at 25 months postoperatively. The patient died 36 months after the surgical intervention. The third patient was a 59-year-old male who presented with left-sided paresis, worsening over the past month. MRI revealed a mass localized to the genu of the corpus callosum and bilateral extensions into the frontal lobes (Figure 3).

The patient was positioned in dorsal decubitus, and a keyhole unilateral anterior interhemispheric approach was used, with the dissection continuing until exposure of the pericallosal arteries. Tumor resection was significantly hindered by extension into the thalamus, limbic structures, and corona radiata. Transcranial and direct stimulation electrophysiology was used for this patient. The immediate postoperative period was uneventful, with the patient regaining partial motor function. Postoperative MRI confirms EOR of 74%. One month later, the patient was re-admitted for acute dysarthria and left-sided hemiparesis, which MRI revealed was precipitated by obstructive hydrocephalus. Tumoral extension from the right horn of the lateral ventricle invaded into the foramen of Monro, necessitating the placement of a ventriculoperitoneal shunt. Following the procedure, the patient rapidly recovered and was discharged three days later. Progression is documented at five months follow-up. The patient has second cytoreductive surgery in another clinic and is still alive at the moment of submission of the paper, 19 months after the first surgery. Karnofsky Performance Status (KPS) scores after resection were 100, 90, and 90 for the three patients, respectively. The genetic profiles of the resected tumors for the first two patients are presented in Table 2.

**Table 2:** Genetic profile of the resected tumors. NA: Not available; IDH: Isocitrate dehydrogenase; TERTp: Telomerase reverse transcriptase; EGFR: Epidermal growth factor receptor; MGMT: O(6)-methylguanine-DNA methyltransferase.

	Microsatellite status	Tumor mutational burden	IDH 1/2 mutation	TERTp	CDKN2A	EGFR Amplification	PIK3CA	MGMT methylation
Patient 1	Stable	Low (4 Muts/Mb)	No	No	Loss (p16INK4a and p14ARF)	Yes (A289V - subclonal)	Yes (E545G)	Negative
Patient 2	Stable	Low (0 Muts/Mb)	No	Yes (-146C>T)	Loss (p16INK4a)	Yes (R108K, EGFRvIII)	No	Negative
Patient 3	NA	NA	NA	NA	NA	NA	NA	NA

During preoperative planning, interhemispheric approaches were chosen for all three patients, given the location and size of the tumors. In addition, this approach minimized the destruction of normal cortical tissue. The interhemispheric approach was the least destructive to the normal brain, and this approach facilitated the optimal cytoreductive result in preoperative planning. All patients have received postoperative MRIs within the first 24 hours after surgical resection.

## Discussion

This study presents the results of three patients who underwent keyhole resection of bGGs. We report operative results and patient outcomes for this procedure and highlight the safety and feasibility of performing bGG resection through a minimally invasive keyhole approach for both anterior and posterior tumors. Determining the role of surgical resection in managing patients with grade 4 gliomas has been a core question in neurological oncology, especially since these tumors are infamous for their aggressive infiltration beyond the borders of contrast enhancement, making complete resection challenging. One of the largest studies to date examined 20,705 adult glioblastoma (GBM) patients to conclude that surgical cytoreduction via both gross total and subtotal resection improved overall survival [3]. For the 45-59-year-old patient age group, the mean survival time for the gross total, subtotal, and no resection was 15, 12, and 7 months, respectively. Several other studies observed similar survival benefits in reducing tumor burden prior to initiating chemoradiotherapy [2]. 

The next pressing question is determining the EOR (calculated as [preoperative tumor volume - postoperative tumor volume]/preoperative tumor volume x 100%) to guide treatment and predict outcomes based on operative data. Precise guidelines are needed as resection must be closely balanced against maximizing the KPS score, ensuring EOR does not increase at the expense of neurological outcome and integrity of eloquent structures. EOR interpretation is contingent upon the definition of gross tumor volume (GTV). Most surgical studies that evaluated EOR and survival benefit calculate GTV using its T1-weighted contrast-enhancing area. This definition of EOR ignores T2-FLAIR components, does not include a margin, and, although straightforward from a surgical point of view, can be misleading as even an EOR of 100% leaves behind residual tumor that is not calculated into the RTV. 

There is pronounced variability in EOR values at which significant survival advantage is noted, with a wide range being reported in the literature [2]. In a retrospective study of 500 patients with glioblastoma, Sanai N et al. examined the threshold of efficacy for microsurgical resection of GBM, after which patients received a standard regimen of chemoradiotherapy [9]. They discovered a significant survival advantage with an EOR of as little as 78% and a further positive correlation between EOR and median overall survival, even when EOR was within the 95%-100% range. This latter finding was furthered by Li YM et al. in a 1229 patient GBM study, which aimed to quantify the survival advantage of complete resection (100% of the contrast-enhancing region) versus extensive, but less expansive resection (78%-99% of the contrast-enhancing region) [10]. The median survival time for the former group was 15.2 months (95% CI: 14.1-16.3 months), while the survival time for the latter group was 9.8 months (95% CI: 8.8-10.8 months). 

Definitive high-quality studies demonstrate a positive stepwise relationship between the extent of EOR and survival advantage. However, evaluating EOR without examining the RTV may skew results, as larger tumors will leave behind more tumoral mass than smaller tumors in the context of an identical EOR. Moreover, quantifying EOR via contrast-enhanced T1-weighted MRI may underappreciate the non-contrast-enhancing T2-FLAIR components of glioblastoma, which are infamously difficult to differentiate from vasogenic edema and may even extend beyond the T2/FLAIR enhancement. Resection of T2/FLAIR abnormalities has been shown to confer an additional survival benefit if it can be done safely [10]. 

Grabowski MM et al. retrospectively reviewed 128 patients and found the contrast-enhancing RTV of less than 2.0 cm3 to be a stronger predictor of survival than EOR of 98% [1]. As such, EOR must be interpreted in tandem with RTV as in the previously mentioned study by Li YM et al., the difference in median preoperative T1-contrast-enhancing volumes between the incomplete versus complete resection cohorts was huge, i.e., 20.4 cm3 (46.3 cm3 versus 25.9 cm3, respectively) [10]. Without reporting median EOR and RTV for the incomplete resection cohort, it remains unclear which parameter should be held as the gold standard for the evaluation of resection in select patients. With mounting evidence outlining the benefits of resection, the logical continuation is a further refinement of the procedure through decreasing surgical wound size, minimizing tissue destruction from dissection, reducing brain exploration and retraction, improving postoperative recovery, and implementing early discharge plans. A minimally invasive ’keyhole’ approach is a philosophy that aligns with these goals by minimizing the incision and bone flap size to mitigate the overall impact of the surgical procedure. Keyhole approaches are more technically demanding than classic craniotomies, as a limited surgical corridor makes it more difficult to maintain intraoperative orientation and adequate illumination in the deep-seated field. Nevertheless, advances in navigational systems, microinstruments, full high-definition endoscopic systems, and other equipment supported the realization of this approach to the point where, now, virtually any intracranial pathology can be approached according to a keyhole principle [11,12]. The application of keyhole craniotomies to perform resection of high-grade gliomas has been successful and current studies that validate the feasibility of this approach are presented in Table 3 [8,11-14].

**Table 3:** Studies which utilized a keyhole approach for resection of low and high-grade gliomas. GBM: Glioblastoma; EOR: Extent of resection; LOS: Length of stay.

Author and year	Location of tumour	Number of patients	Median tumor volume in cm^3^(range, if available)	Median total EOR	Median surgery duration (minutes)	Median LOS (days)
Conner AK et al. (2018) [12]	Temporal lobe	52 (35 GBM)	41 (1.7-154.4)	95%	250 awake/224 asleep	4
Burks JD et al. (2018) [11]	Frontal lobe	48 (22 GBM)	20.6 (3.5-137.6)	98%	213 awake/220 asleep	3
Conner AK et al. (2017) [13]	Occipital lobe	8 (7 GBM)	28 (9-41)	96%	162	6
Sughrue ME et al. (2016) [14]	Insular lobe	20 (5 GBM)	-	99%	215	3
Dadario NB et al. (2022) [8]	Corpus callosum	70	-	>95% EOR in 63 cases	-	<5

Current literature suggests excellent EOR and early discharge times are possible without higher rates of adverse events. The postoperative hospitalization time of five days of our three patients is consistent with LOS reported in these studies. Sughrue ME et al. argue that most life-threatening complications associated with elective brain tumor surgery occur within a few hours after surgery [15]. In their study, 70.6% of patients who underwent surgical cytoreduction of high-grade gliomas via a keyhole approach benefitted from early discharge (within one or two days after the procedure) with a readmission rate of 4.9%, thus demonstrating the viability of leveraging safe early discharge in patients who undergo minimally invasive high-grade glioma resection. The three patients presented in our study had bGG, defined by their invasion through the corpus callosum into the contralateral hemisphere. This is also why they were traditionally considered inoperable via standard transcortical approaches. Bihemispheric involvement in grade 4 glioma patients has an estimated incidence of 2.2-3.8%, and this rarity is mirrored by an equally dismal median survival time of just six months [6,7,16]. After doing a biopsy to confirm the diagnosis, the conventional treatment for bGG was radiation with chemotherapy. Surgical resection of gliomas involving the corpus callosum has historically been discouraged by the neurosurgical community out of fear of precipitating profound akinetic mutism, abulia, and other devastating neurologic sequelae. Despite being deemed inoperable, some persisted in attempting to refine surgical techniques to overcome technical challenges and achieve the same cytoreductive survival benefit that has already been well established for traditional grade 4 gliomas. Safe resection could also address patients' poor quality of life on palliative therapy. Substantial bihemispheric tumoral burden and the associated edema resistant to bifrontal radiation results in rapid progression to abulia and akinesia, making some question how much worse surgery could be [4,6]. The grim reality of patient suffering can be inferred from Burks JD et al. study, where out of the 42 patients with butterfly glioblastomas who were offered to be enrolled into the study and undergo experimental surgical resection (with extensive counseling on high risks), 40 patients (95%) agreed to surgery [4].

Resection of anterior bGG has become safer due to advances in intraoperative task-based cortical mapping and subcortical monitoring, as well as improvements in surgical techniques, namely sparing the default mode network with its connections and preserving the caudate, cingulum, septal nuclei, and the anterior cerebral artery [4,6]. Intraoperative MRI, 3D ultrasound-guided resection, 5-aminolevulinic acid (5-ALA) for real-time tumor identification, and functional neuronavigation systems are other examples of modern tools that improve safety and EOR in bGG. For posterior bGG, a transcortical subpial approach with early identification of the tumor and internal circumferential debulking has been successfully applied [7]. In our patients, surgery was offered because the tumors could be reached via an interhemispheric approach, with good angulation providing adequate space to ensure safe EOR. A commonly cited problem is the low incidence of bGG, resulting in a paucity of high-powered studies examining optimal surgical management. Some of the current reports which examined the survival advantage of resecting bGG are presented in Table 4 [4-8,16-18]. 

**Table 4:** Studies examining the survival benefit of surgical resection of butterfly grade 4 glioma. EOR: Extent of resection; LOS: Length of stay.

Author and year	Number of patients	Median tumor volume in cm^3^(range if available)	GTR (EOR>95%)	Median total EOR	Median LOS (days)	Median survival (months)
Dayani F et al. (2018) [6]	14	43.7 (33.8-70.2)	-	83.0% (44.2%-100%)	-	14.06
Opoku-Darko M et al. (2017) [7]	9	62.9 ± 5.4	>98% in five patients; <98% in four patients	-	12	7.8
Burks JD et al. (2017) [4]	40	-	84%	100%	-	5
Chaichana KL et al. (2014) [5]	29	45.1 (28.5-75.3)	-	61.40%	8	7
Dziurzynski K et al. (2012) [16]	11	40.5 (0.8-178.1)	54%	100 (24.7-100)	-	8.8
Franco P et al. (2021) [17]	25	59.4	GTR in 32%	-	-	8.6
Boaro A et al. (2021) [18]	26	47.5	11%	72.3% (58.3-82.1)	6.7	11.5
Dadario NB et al. (2022) [8]	52	-	GTR in >90%	-	-	10

All studies performed volumetric analysis on MRI with T1-weighted imaging with gadolinium contrast. For all studies, surgery was not offered to patients with a KPS score of under 70, with most studies enrolling patients with a KPS score of 80-90. None of the studies included neuropsychological assessment, which is significant as in their 40-patient cohort, Burks JD et al. noted their patients often presented with significant frontal lobe-based cognitive problems, which nonetheless did not preclude them from normal day-to-day function [4]. In our three patients, no neuropsychological assessment was done, but preoperative KPS was very good, as they were all normally functioning just a few weeks before diagnosis. Recent metanalyses evaluate the feasibility of surgical resection of bGG, suggesting that surgical resection is associated with improved survival [19,20]. Even more importantly, there were no statistically significant differences between postoperative complication rates between the resections group vs. the biopsy alone group [20]. In these metanalyses, the longest survival length was the one reported by Dziurzynski K et al. of 1018 days (2.8 years) [16]. One of our patients has a survival of more than three years (1109 days). Chawla S et al. found that resection surgery of butterfly glioblastomas was associated with longer overall survival compared with biopsy alone, and the effect of surgery persisted in both >80% and <80% EOR subgroups [20]. Interestingly, the patient no. 3 in our case series with an EOR of only 74% on first surgery is alive at the moment of writing the article, 19 months after the first surgery. There is a general tendency to operate on larger tumors, as the indication is the rapid deterioration because of the mass effect of the tumor [17,18,20]. The potential immediate danger of mass effect over the patient indicates the surgical treatment, over biopsy and chemoradiation, as on very large tumors, these adjuvant therapies would have very little effect in a short time. The smaller volume tumors tend to be biopsied. However, data suggest that the better the EOR, the better the survival prognosis for the patients. Surgery should be considered for all patients if feasible. None of our patients was in danger of mass effect. Compared to the metanalyses, our patients would correspond more to the biopsy group than to the surgical group. Nevertheless, the very good survival rates for our small case series add up to the general trend of advocating for surgery for these delicate patients.

## Conclusions

We add up three more cases to the few reported bGG operated through the keyhole approach, and we also report a long-term survivor for bGG. The application of all current medical knowledge, neuroanesthesia, neuroanatomy, neurosurgery, and the intraoperative implementation of other modern technology already allows for the performance of surgical interventions that are still deemed impossible or with no benefit in many neurosurgical centers around the world. The favorable clinical outcome obtained in our patients using a keyhole approach for the resection of anterior and posterior bGG adds to existing evidence supporting the role of minimally invasive cytoreduction in the treatment strategy for this pathology. In the case of the three patients presented here, surgical resection was technically feasible with minimal injury to the normal cortex. The results presented herein suggest a favorable impact of surgery on both the survival and quality of life of patients with bGG. However, more high-powered studies are needed to explore this further.

## References

[1] Grabowski MM, Recinos PF, Nowacki AS, Schroeder JL, Angelov L, Barnett GH, Vogelbaum MA (2014). Residual tumor volume versus extent of resection: predictors of survival after surgery for glioblastoma. J Neurosurg.

[2] Lacroix M, Abi-Said D, Fourney DR (2001). A multivariate analysis of 416 patients with glioblastoma multiforme: prognosis, extent of resection, and survival. J Neurosurg.

[3] Noorbakhsh A, Tang JA, Marcus LP (2014). Gross-total resection outcomes in an elderly population with glioblastoma: a SEER-based analysis. J Neurosurg.

[4] Burks JD, Bonney PA, Conner AK (2017). A method for safely resecting anterior butterfly gliomas: the surgical anatomy of the default mode network and the relevance of its preservation. J Neurosurg.

[5] Chaichana KL, Jusue-Torres I, Lemos AM (2014). The butterfly effect on glioblastoma: is volumetric extent of resection more effective than biopsy for these tumors?. J Neurooncol.

[6] Dayani F, Young JS, Bonte A (2018). Safety and outcomes of resection of butterfly glioblastoma. Neurosurg Focus.

[7] Opoku-Darko M, Amuah JE, Kelly JJ (2018). Surgical resection of anterior and posterior butterfly glioblastoma. World Neurosurg.

[8] Dadario NB, Zaman A, Pandya M, Dlouhy BJ, Gunawardena MP, Sughrue ME, Teo C (2022). Endoscopic-assisted surgical approach for butterfly glioma surgery. J Neurooncol.

[9] Sanai N, Polley MY, McDermott MW, Parsa AT, Berger MS (2011). An extent of resection threshold for newly diagnosed glioblastomas. J Neurosurg.

[10] Li YM, Suki D, Hess K, Sawaya R (2016). The influence of maximum safe resection of glioblastoma on survival in 1229 patients: Can we do better than gross-total resection?. J Neurosurg.

[11] Burks JD, Conner AK, Bonney PA (2018). Frontal keyhole craniotomy for resection of low- and high-grade gliomas. Neurosurgery.

[12] Conner AK, Burks JD, Baker CM (2018). Method for temporal keyhole lobectomies in resection of low- and high-grade gliomas. J Neurosurg.

[13] Conner AK, Baker CM, Briggs RG (2017). A technique for resecting occipital pole gliomas using a keyhole lobectomy. World Neurosurg.

[14] Sughrue ME, Othman J, Mills SA, Bonney PA, Maurer AJ, Teo C (2016). Keyhole transsylvian resection of infiltrative insular gliomas: technique and anatomic results. Turk Neurosurg.

[15] Sughrue ME, Bonney PA, Choi L, Teo C (2015). Early discharge after surgery for intra-axial brain tumors. World Neurosurg.

[16] Dziurzynski K, Blas-Boria D, Suki D, Cahill DP, Prabhu SS, Puduvalli V, Levine N (2012). Butterfly glioblastomas: a retrospective review and qualitative assessment of outcomes. J Neurooncol.

[17] Franco P, Delev D, Cipriani D (2021). Surgery for IDH1/2 wild-type glioma invading the corpus callosum. Acta Neurochir (Wien).

[18] Boaro A, Kavouridis VK, Siddi F (2021). Improved outcomes associated with maximal extent of resection for butterfly glioblastoma: insights from institutional and national data. Acta Neurochir (Wien).

[19] Chojak R, Koźba-Gosztyła M, Słychan K, Gajos D, Kotas M, Tyliszczak M, Czapiga B (2021). Impact of surgical resection of butterfly glioblastoma on survival: a meta-analysis based on comparative studies. Sci Rep.

[20] Chawla S, Kavouridis VK, Boaro A (2022). Surgery vs. biopsy in the treatment of butterfly glioblastoma: a systematic review and meta-analysis. Cancers (Basel).

